# Cognitive impairment in hemodialysis patients: Implementation of cut-off values for the Montreal Cognitive Assessment (MoCA)-test for feasible screening

**DOI:** 10.1371/journal.pone.0184589

**Published:** 2017-10-10

**Authors:** Susanne Angermann, Marcus Baumann, Dominik Steubl, Georg Lorenz, Christine Hauser, Yana Suttmann, Anna-Lena Reichelt, Robin Satanovskij, Franziska Sonntag, Uwe Heemann, Timo Grimmer, Christoph Schmaderer

**Affiliations:** 1 Department of Nephrology, Klinikum rechts der Isar, Technische Universität München, Munich, Bavaria, Germany; 2 Department of Nephrology, Klinikum Ansbach, Friedrich-Alexander-Universität Erlangen-Nürnberg, Erlangen-Nuremberg, Bavaria, Germany; 3 Department of Psychiatry and Psychotherapy, Klinikum rechts der Isar, Technische Universität München, Munich, Bavaria, Germany; Biomedical Research Foundation, UNITED STATES

## Abstract

**Objective:**

Reliable identification of cognitive impairment in hemodialysis patients is of utmost importance, as it is associated with poor outcomes including dialysis withdrawal and death. High prevalence of cognitive impairment has been demonstrated in several studies using brief screening instruments or neuropsychological test batteries. However, the relevance of cognitive impairment as well as the accuracy of screening procedures have never been studied in this patient population.

**Methods:**

151 chronic hemodialysis patients (mean age 65.78 ± 14.88 years, 73,5% male) underwent cognitive testing under standardized conditions by the Montreal Cognitive Assessment (MoCA) and, in a second step, the Clinical Dementia Rating scale (CDR), an international standard to measure the severity of dementia. For calculating MoCA cut-off values on the basis of the CDR global score, receiver operator characteristics (ROC) analysis and c-statistic were applied.

**Results:**

49.0% of patients were categorized as 0.5 in the CDR global with memory being the predominantly affected domain (47.7% of patients scored ≥ 0.5). Youden’s Index led to a threshold of 23.5 points for the MoCA test for optimal differentiation between cognitively normal (CDR global < 0.5) and impaired patients (CDR global ≥ 0.5) based on a sensitivity of approximately 99% and a specificity of approximately 74%.

**Conclusion:**

Interference of cognitive impairment with patients’ independence and daily life was shown using the CDR for the first time in hemodialysis patients. A MoCA score of 23.5 points turned out as optimal threshold to differentiate between patients with and without functional impairment in the CDR, thereby paving the way for implementation of the MoCA test as a quick and thus highly feasible screening instrument for periodic testing in clinical routine.

## Introduction

Several studies demonstrated high prevalence of cognitive impairment in hemodialysis patients [1–3] as well as its potential adverse effects on management and outcome including interference with informed decision making, capacity for self-care and adherence to medical, fluid and dietary instructions [4–5]. Moreover, dementia is associated with disability, hospitalization and an approximately 2-fold increased risk of both dialysis withdrawal and death [6–8]. Thus, periodic screening is needed to identify patients with relevant cognitive impairment in order to improve their clinical care as well as to reduce health care costs. In this context, a short screening instrument is the most resource-efficient option, which has to be precise in identifying impaired patients as well as those in need for further evaluation. In previous studies in hemodialysis patients the Montreal Cognitive Assessment (MoCA) test [9], the Mini-Mental State Examination (MMSE) [10] or the 3MS [11] have been applied using established cut-offs. Only one study validated a short screening instrument, the MoCA, identifying a cut-off of 24 points to be more suitable to detect cognitive impairment compared to the established value of 26 points [12].

According to the Diagnostic and Statistical Manual of Mental Disorders (DSM)-IV [13] and the International Classification of Diseases (ICD)-10 [14] guidelines the diagnosis of dementia is based on the presence of cognitive impairment and consequently an impairment of daily activities and independence. The Clinical Dementia Rating scale (CDR) is an international standard to assess the overall severity of dementia taking both cognitive symptoms and impairment in activities of daily living into account by interviewing the patient and his informant or caregiver. Despite its important role in evaluating the degree of cognitive impairment independent from the underlying condition, the CDR has never been applied in hemodialysis patients before.

Hence, the aims of our study were to examine the overall degree of dementia in this patient population for the first time using the CDR and to reevaluate cut-off values for the MoCA test to distinguish cognitively normal and impaired hemodialysis patients.

## Materials and methods

### Patients, inclusion and exclusion criteria

**Fig 1:**
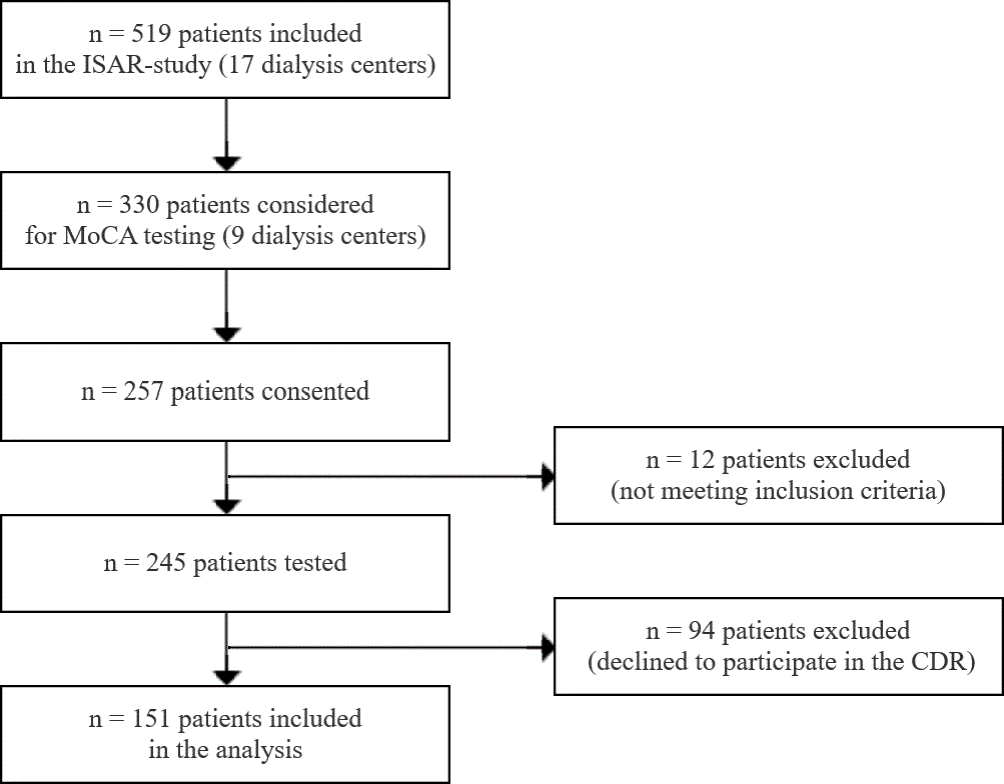
Flowchart–data acquisition process within the ISAR study. ISAR: rISk strAtification in end-stage renal disease; MoCA: Montreal Cognitive Assessment; CDR: Clinical Dementia Rating scale.

This study is part of the ISAR-study, which is registered at ClinicalTrials.gov (identifier number: NCT01152892, URL: https://clinicaltrials.gov/ct2/show/NCT01152892?term=ISAR&rank=2). ISAR is an observational study to evaluate the use of non-invasive markers of autonomic function and micro- and macrocirculation to predict mortality and cardiovascular end points in end stage renal disease (ESRD) patients. The complete study protocol in reference to the STROBE guidelines was published in BMC Nephrology in 2016 [15]. Evaluation of cognitive function was part of a sub study and therefore cognitive testing was performed only in 9 centers of a total of 17 centers taking part in the ISAR study. In order to maximize participation, patients were allowed to decide for themselves, which examinations to undergo. As a result, a total of 151 patients were tested by both, MoCA and CDR. Fig 1 depicts the data acquisition process throughout this sub study of the ISAR study.

The study protocol as well as an amendment specifically covering cognitive testing by both MoCA and CDR were approved by the committees of the Faculty of Medicine of the Technische Universität München and of the medical chamber of Bavaria. All clinical investigations were conducted in accordance with the principles of the Declaration of Helsinki, sixth revision. Written informed consent was obtained from all participants before any study specific procedure.

Patients were recruited from nine hemodialysis centers located in Munich and surrounding area between June 2011 and July 2013. Inclusion criteria were age of 18 years or older and dialysis vintage of at least three months. General exclusion criteria were systemic infection, electrolyte disorder, malignancy and pregnancy. Conditions that may interfere with cognitive testing were also defined as exclusion criteria, such as not speaking the local language proficiently, motoric disorders of the dominant hand, aphasia or amaurosis.

Demographics, comorbidities and dialysis vintage were obtained from medical reports or by patient interviews, the ultrafiltration volume from the protocol of the patients’ most recent dialysis session. Kt/V, which serves as an equivalent for the dialysis efficacy (urea clearance), was derived from the most recent laboratory examination of the corresponding dialysis center.

### Cognitive testing

The MoCA test was applied as a short screening instrument for cognitive function [9], which covers the domains attention and concentration, memory, orientation, language, visuoconstructional skills, conceptual thinking, calculations and executive functions. A maximum of 30 points is attainable, whereas a score of 26 is used as a cut-off value between normal and pathological patterns based on a comparison between patients with dementia due to Alzheimer’s disease and healthy controls. The MoCA was chosen as cognitive testing instrument in this study for feasibility reasons and because it explicitly covers executive functions, which appear to be impaired early in hemodialysis patients [3, 16]. Other tests, for example the MMSE, do not include evaluation of this cognitive domain. Cognitive testing was performed on a midweek dialysis day and under standardized conditions (before dialysis in a separate room), as published previously [17]. All patients were tested in German using the original version of the MoCA test. A total of four raters performed the MoCA testing throughout this study, all intensively trained by the same professional prior to performing patient assessments.

The CDR was chosen serving as equivalent for the overall severity of cognitive impairment and dementia. The instrument covers six cognitive and functional domains, which are memory, orientation, judgment and problem solving, community affairs, home and hobbies as well as personal care. Each domain is rated on a 5-point scale with 0 meaning no impairment, 0.5 meaning questionable impairment, until 3 meaning maximum impairment. The six domain ratings are integrated into one overall score, which also ranges from 0 (no dementia) to 3 (severe dementia), which is referred to as CDR global. The CDR SOB (Sum Of Boxes) on the other hand is derived by adding the scores from all six individual categories. Interviews of patients’ informants or caregivers were carried out face-to-face or via telephone, whereby most of the interviews were conducted by telephone due to feasibility reasons. All 151 patients including their respective informants or caregivers were examined by the same person, who was trained comprehensively and supervised by an experienced CDR rater.

### Statistical analyses

SPSS 21.0 software (www.spss.com) was used for all statistical tests.

For calculations including MoCA scores, MoCA raw values were applied (not adjusted for educational level), based on the assumption that one extra point added to the total score, as proposed by the authors, is not sufficient, to adjust for this very important factor. This reflects our general approach for all analyses including the MoCA score within the ISAR-study.

Demographic and clinical characteristics were obtained by means of descriptive statistics.

In order to rule out a systematic selection of those patients, who underwent cognitive evaluation by the MoCA but not by the CDR compared to those, who received both testing modalities, a Mann-Whitney-test for the MoCA raw value and a Chi-Square-test for educational level were carried out.

In a second step CDR global as well as CDR SOB values were compared to MoCA raw values of patients using scatterplots to check validity of data. Spearman rank correlation analyses with MoCA raw, CDR global and SOB values as well as the CDR memory item were performed.

In order to establish appropriate cut-off values for the MoCA test in hemodialysis patients to differentiate between normal (CDR global = 0) and cognitively impaired (CDR global ≥ 0.5), receiver operator characteristics (ROC) analysis and c-statistic were applied. Youden’s Index was used to determine threshold values.

## Results

### Patients

151 patients with a mean age of 65.78 ± 14.88 years were included, 73.5% of them male. 62.3% of patients had formal education of 12 years or less. Further comorbidities as well as the most important hemodialysis-associated parameters are provided in Table 1.

**Table 1:** Demographics, comorbidities and dialysis data.

**Demographics**	
Age (years), mean ± SD	65.78 ± 14.88
Sex m/f, *n* (%)	111 (73.5)/40 (26.5)
Educational level ≤ 12 years, *n* (%)	94 (62.3)
**Comorbidities**	
Arterial hypertension, *n* (%)	143 (94.7)
Diabetes mellitus, *n* (%)	59 (39.1)
Hypercholesterolemia, *n* (%)	102 (67.5)
Current smokers, *n* (%)	33 (21.9)
Prevalence of CVD, *n* (%)	87 (57.6)
**Dialysis data**	
Dialysis vintage (months), mean ± SD	56.63 ± 55.42
Kt/V, mean ± SD	1.49 ± 0.32
Ultrafiltration volume (liters), mean ± SD	1.74 ± 1.16

SD: standard deviation; m/f: male/female; CVD: cardiovascular disease: history of myocardial infarction, stroke, peripheral artery disease or other kinds of atherosclerosis; arterial hypertension: regular intake of antihypertensive medication; hypercholesterolemia: regular intake of statins or total cholesterol levels > 200 mg/dl; dialysis vintage: cumulative time of patients requiring dialysis.

### Systematic drop-out

The comparison of patients that underwent both the MoCA and the CDR assessment to those patients that refrained from participating in the CDR assessment revealed a numerical mean difference of 0.25 points of MoCA raw values, which seems negligible. The between-group-comparison of MoCA raw values and educational level did not attain statistical significance (Table 2).

**Table 2:** Comparison—patients with MoCA and CDR versus MoCA only.

	MoCA and CDR (n = 151)	MoCA only (n = 91)	p-value
**MoCA raw score (mean ± SD)**	24.09 ± 3.93	23.84 ± 3.78	0.527 (Mann-Whitney)
**Education ≤ 12 years, n (%)**	95 (62.9)	57 (62.6)	0.685 (Chi-Square)

CDR: Clinical Dementia Rating scale; MoCA: Montreal Cognitive Assessment.

### Evaluation of cognitive function

Applying pre-established thresholds, MoCA score of patients would have been considered as abnormal (mean of 24.09 ± 3.93) with the subcategory memory function/recall predominantly affected (mean of 2.74 ± 1.77 points; maximum attainable points: 5).

Patients’ performance in all individual CDR domains as well as their CDR global scores are provided in Table 3.

**Table 3:** Evaluation of cognitive function–CDR.

	0	0.5	1	2	3
**memory, n (%)**	79 (52.3)	34 (22.5)	37 (24.5)	1 (0.7)	0 (0.0)
**orientation, n (%)**	146 (96.7)	4 (2.6)	1 (0.7)	0 (0.0)	0 (0.0)
**judgement and problem solving, n (%)**	121 (80.1)	24 (15.9)	6 (4.0)	0 (0.0)	0 (0.0)
**community affairs, n (%)**	128 (84.8)	21 (13.9)	2 (1.3)	0 (0.0)	0 (0.0)
**home and hobbies, n (%)**	132 (87.4)	15 (9.9)	4 (2.6)	0 (0.0)	0 (0.0)
**personal care, n (%)**	138 (91.4)		12 (7.9)	1 (0.7)	0 (0.0)
**CDR global, n (%)**	75 (49.7)	74 (49.0)	2 (1.3)	0 (0.0)	0 (0.0)

CDR: Clinical Dementia Rating scale.

Memory function was the CDR domain predominantly affected: approximately 25% with 0.5 points and approximately 25% with 1.0 points. In the domains judgement and problem solving as well as community affairs almost 20% of patients were classified as impaired.

**Fig 2:**
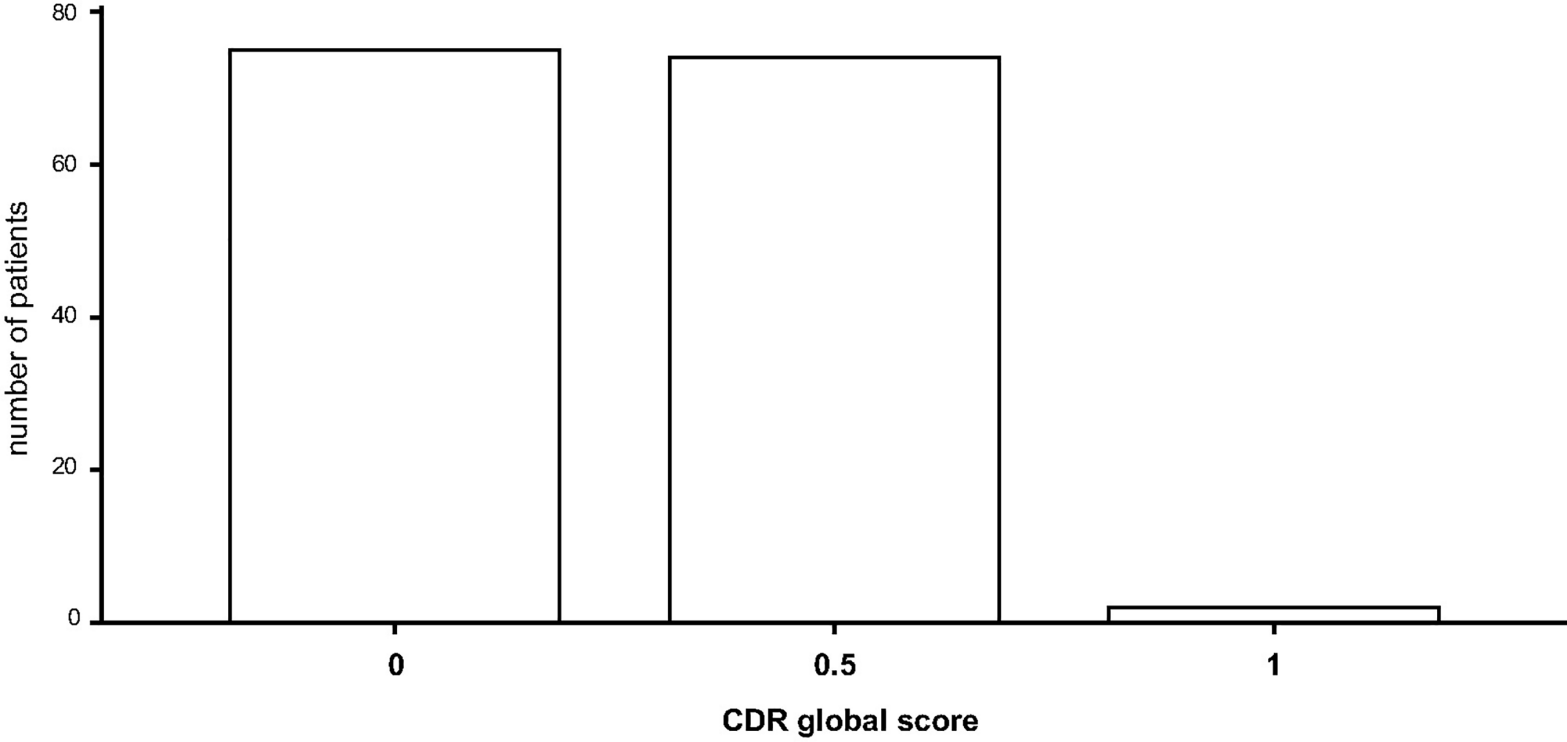
Evaluation of cognitive function (CDR): memory. CDR: Clinical Dementia Rating scale.

**Fig 3:**
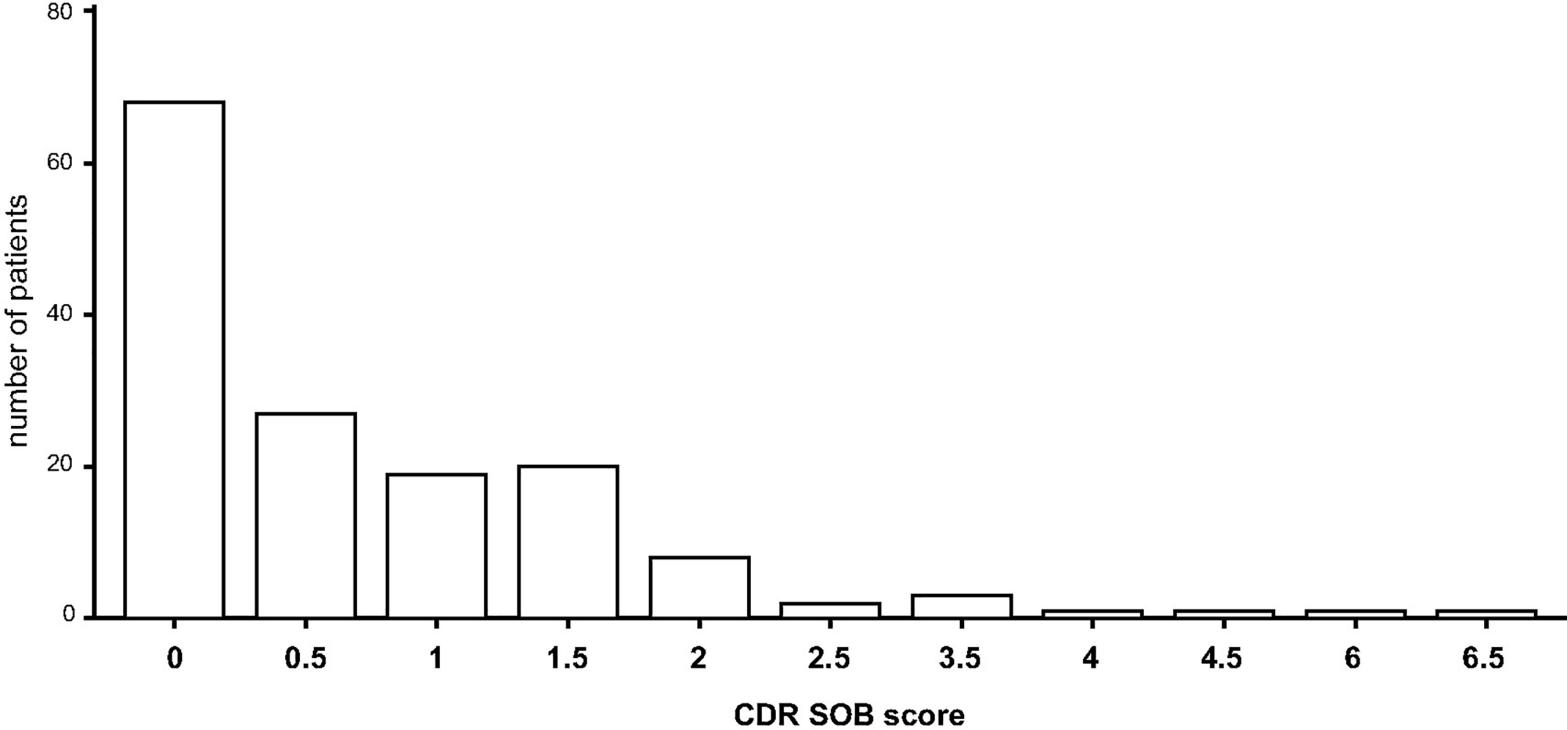
Evaluation of cognitive function: CDR global. CDR: Clinical Dementia Rating scale.

**Fig 4:**
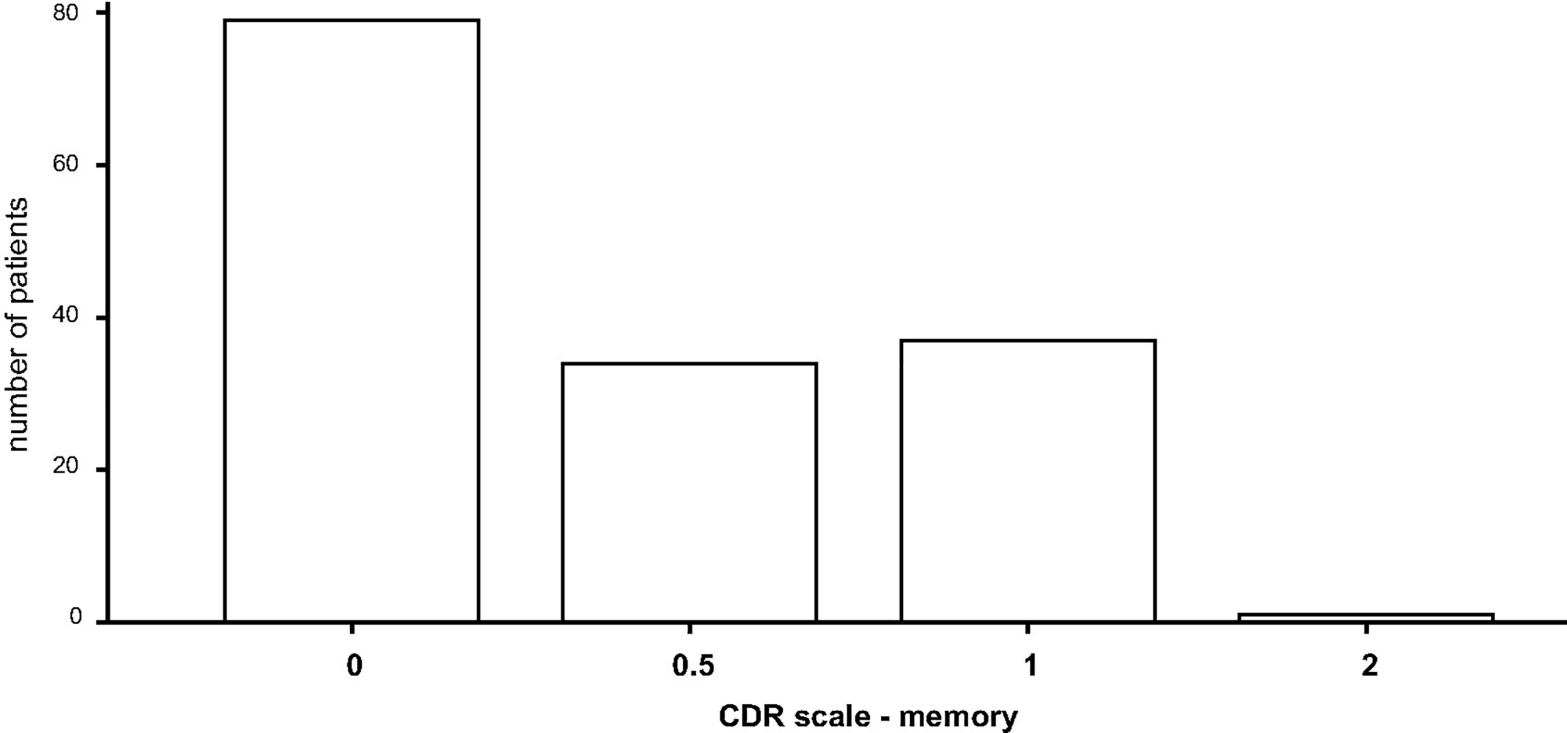
Evaluation of cognitive function: CDR SOB. CDR: Clinical Dementia Rating scale; SOB: Sum Of Boxes.

With respect to CDR global approximately 50% of patients scored 0 implying no impairment, whereas the other half scored 0.5 implying mild cognitive impairment. The average CDR SOB score was 0.762 ± 1.09. Patients’ average scores for CDR global, CDR SOB, and the CDR domain memory are depicted in bar diagrams (Figs 2–4).

### Data validity check

Consistency between MoCA and CDR results was illustrated by box plot diagrams (see S1 and S2 Figs). Correlations between MoCA raw values and scores in the CDR domain memory as well as between CDR global and CDR SOB scores were highly significant (p < 0.001 for memory (rho = -0.711) as well as CDR global (rho = -0.605) and CDR SOB (rho = -0.600)).

### MoCA cut-off values for relevant impaired cognition

**Fig 5:**
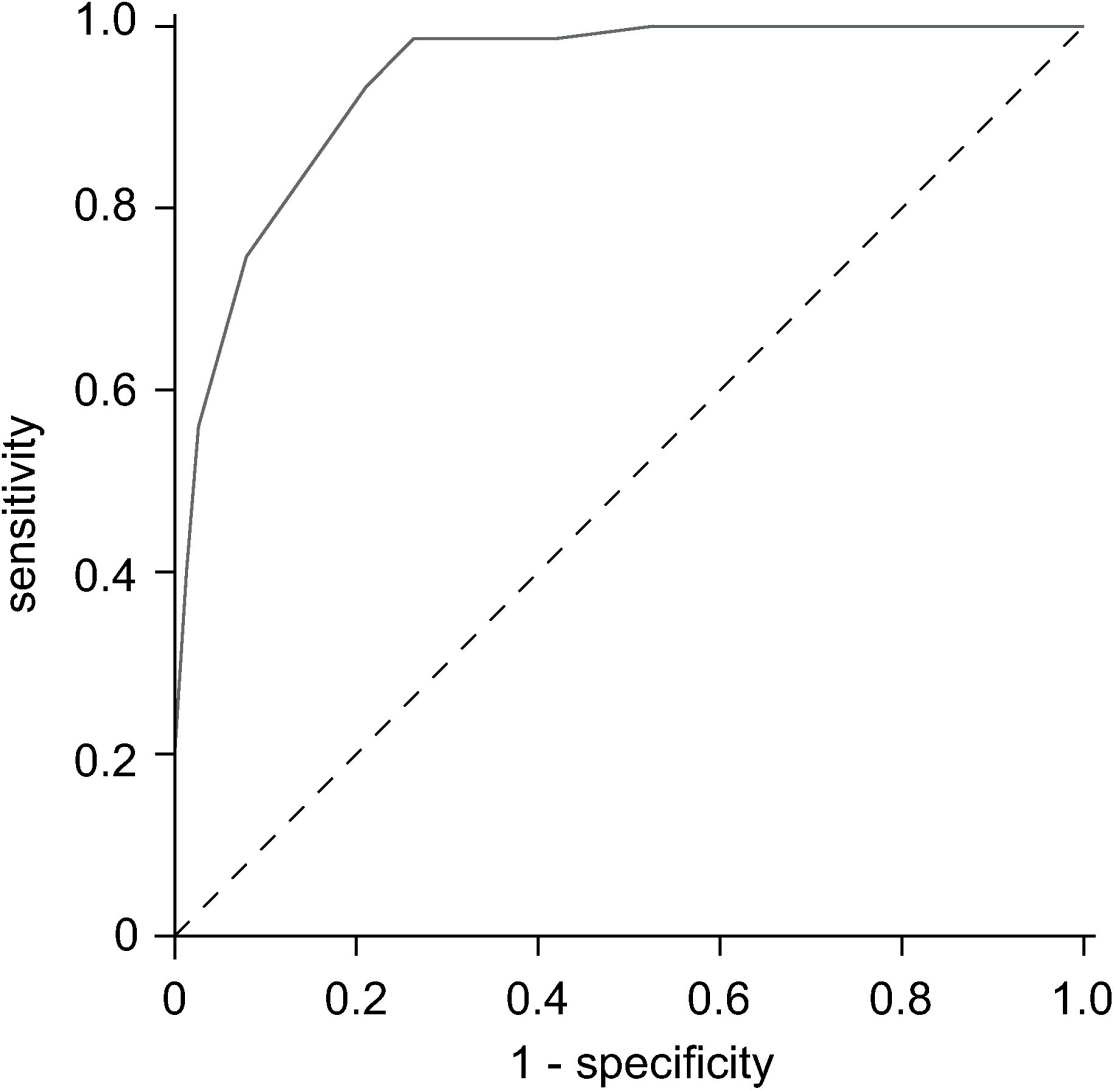
ROC curve of MoCA test scores to differentiate between CDR global of 0 and ≥ 0.5. ROC: Receiver Operator Characteristics; CDR: Clinical Dementia Rating scale.

Fig 5 displays the ROC curve for the MoCA raw value to differentiate between CDR global scores of 0 and ≥ 0.5. For a MoCA raw value of 21.5 points sensitivity to detect cognitively impaired patients (CDR global ≥ 0.5) reached 100% (specificity = 47%). For a MoCA raw value of 28.5 points on the other hand, specificity not to misclassify any cognitively normal (CDR global = 0.5) patients as cognitively impaired reached 100% (sensitivity = 20%). Youden’s Index led to a threshold of 23.5 points for the MoCA test based on a sensitivity of approximately 99% and a specificity of approximately 74%.

## Discussion

The current study aimed to identify clinically relevant cognitive impairment in hemodialysis patients by using a standardized interview (the CDR) in a cohort of 151 hemodialysis patients. On the basis of the CDR global score the following cut-off values for the MoCA test were derived: A MoCA score of < 22 is associated with cognitive impairment in daily life with absolute certainty, a MoCA score of > 28 rules out cognitive impairment in daily life with absolute certainty, whereas scores of 22–28 should be considered as equivocal. A MoCA score of 23.5 points turned out to be the best cut-off to differentiate between cognitively normal and cognitively impaired hemodialysis patients.

The main novelty of our study was to evaluate hemodialysis patients by the CDR, which is the international standard for the documentation of the overall severity of dementia. Memory function turned out to be the predominantly affected individual CDR domain with over 50% of patients categorized as either affected by mild cognitive impairment or mild dementia. Concerning overall scores, 50.3% of patients reached at least a CDR global of 0.5 (mild cognitive impairment), which indicates incipient interference with patients’ independence and daily life activities. To establish the diagnosis of dementia DSM-IV as well as ICD-10 guidelines require the latter criterion to be fulfilled apart from proven impairment in memory function and at least one other domain of cognitive function. Thus, this was the first time to show evidence for and highlight relevance of cognitive impairment in hemodialysis patients at the same time. To the best of our knowledge no other study covered this important aspect in hemodialysis patients so far.

On the basis of the CDR global score new cut-off values for the MoCA test in hemodialysis patients were established with the optimal score to differentiate between cognitively normal and impaired patients having been determined to be 23.5 points. This cut-off value corresponds almost exactly to the one found by Tiffin-Richards et al. in 2014 [12]. In this study 43 hemodialysis patients with an average age of 58 years, were assessed with the MoCA, the MMSE and a detailed neuropsychological test battery, which was used as the gold standard. A MoCA cut-off of 24 points identified patients with cognitive impairment with a sensitivity of 77% and a specificity of 79%. The MMSE on the other hand only discriminated weakly between groups.

One limitation of the present study is a possible recruitment bias. Patients who already noticed cognitive impairment might have refrained from participating. Therefore, patients with moderate to severe cognitive impairment may not have been included. In addition, the MoCA cut-offs established in our study cohort might also be influenced by other factors such as demographics or comorbidities. However, post-hoc non-parametric partial correlation controlling for age and educational level yielded a significant association between MoCA raw and CDR global scores (p < 0.001, rho = -0.660). Furthermore, it is noteworthy in this context that the cut-off of 23.5 points calculated in our study was also identified in hemodialysis patients in another study, cited above [12]. Nevertheless, our finding needs to be replicated in independent samples.

Whether the new MoCA cut-off is suitable to predict adverse outcomes including mortality cannot be concluded from the current study, but will be subject of future research.

This study evaluated hemodialysis patients using the CDR for the first time and found 50.3% of patients classified as at least mildly impaired, which once again underlines the importance of cognitive impairment as a relevant prognostic factor for both clinical outcomes and mortality in this patient population. We were able to establish thresholds for the MoCA test to distinguish cognitively normal and cognitively impaired hemodialysis patients. Thus, periodic screening to identify cognitively impaired or potentially impaired patients is now more feasible and can be implemented in clinical routine with relative ease.

## Supporting information

S1 Fig Consistency of data–CDR global and MoCA raw values. MoCA: Montreal Cognitive Assessment; CDR: Clinical Dementia Rating scale.
(EPS)

S2 Fig Consistency of data–CDR SOB and MoCA raw values. MoCA: Montreal Cognitive Assessment; CDR: Clinical Dementia Rating scale; SOB: Sum Of Boxes.
(EPS)

S1 Data SPSS file with original data used for analyses within this publication. (SAV)
